# Quantitative trait loci (QTL) mapping for intermittent drought tolerance in BRB 191 × SEQ 1027 Andean Intragene cross recombinant inbred line population of common bean (*Phaseolus vulgaris* L.)

**DOI:** 10.5897/AJB2019.16768

**Published:** 2019-05-22

**Authors:** M. Nabateregga, C. Mukankusi, B. Raatz, R. Edema, S. Nkalubo, B. M. E. Alladassi

**Affiliations:** 1 College of Agricultural and Environmental Science, Department of Agricultural Production, Makerere University, P. O. Box 7062 Kampala, Uganda; 2 International Centre for Tropical Agriculture (CIAT), P. O. Box 6247 Kampala, Uganda; 3 CIAT-International Centre for Tropical Agriculture, Cali, Colombia; 4 National Crops Resources Research Institute, Namulonge, P. O. Box 7084, Kampala, Uganda

**Keywords:** Common bean, drought tolerance, single nucleotide polymorphism, quantitative trait loci

## Abstract

Drought is a major constraint of common bean (*Phaseolus vulgaris* L.) production in Uganda where irrigation for the crop is very uncommon. This study aimed to identify quantitative trait loci (QTLs) underlying drought tolerance in 128 F5 RILs derived from an Andean intra-gene cross between drought-tolerant SEQ 1027 and BRB 191. Eighteen traits were evaluated under drought stress and non-stress conditions in the field for 2 years and in the greenhouse for 1 year, respectively. A linkage map spanning 486.29 cM was constructed using 53 single nucleotide polymorphic markers (SNP) markers obtained from the KASP genotyping assay. Eleven consistent QTLs were detected on five linkage groups at a threshold of Logarithm of Odds (LOD) ≥ 3.0. Four QTLs were constitutive, seven were adaptive and were associated with 100 seed weight, grain yield, chlorophyll content, harvest index, dry weight of leaf and stem biomass and yield production efficiency. The QTL associated with a 100 seed weight (*sw3.1^BS^*) was the most consistent with the highest percentage of variation explained (21%). Co-localization of five drought-related factors QTLs was detected on pv10 suggesting pleiotropic effects on this chromosome. Identification of molecular markers closely linked to the QTLs identified in this study will facilitate marker assisted breeding for drought tolerance.

## INTRODUCTION

Common bean is consumed by large numbers of the poor in Africa (Singh and Munoz, 1999). The crop provides protein, complex carbohydrates, and valuable micronutrients for more than 300 million people in the tropics and is a staple crop for over 200 million people in sub-Saharan Africa (Akibode, 2011). The crop therefore plays an important role in mitigating protein malnutrition and micronutrient deficiencies in regions where their effects are prevalent. Furthermore, epidemiological studies also have shown that a regular diet with beans brings great benefits to health due to the fact that it reduces the risk of developing cancer, diabetes and heart disease (Guajardo-Flores et al., 2013). The importance of common bean will increase in the future especially in tropical Africa where the demand is even likely to increase as the human population increases (Wortmann, 2006; Baudoin and Mergeai, 2001). In East Africa, beans are primarily grown by the smallholder farmers, especially women, for home consumption, while any excess production is sold at the market (Spence, 2006).

Drought has been recognized as one of the most important bean production constraint affecting 60% of bean production in drought prone areas worldwide. On-farm yields of beans in East Africa and Uganda in this case, have remained stagnant (0.5 to 0.6 Mt ha^-1^) (Sibiko, 2012; Kalyebara and Buruchara, 2008), a case attributed to a multitude of biotic and abiotic constraints affecting bean production under low input systems. Rainfall in the country is highly variable and unpredictable in most parts of bean production, and yet most of the country’s agriculture is rain-fed; this has made major dry bean yield losses inevitable (NEMA, 2001; Okonya et al., 2013). Effects of drought in major agricultural areas are expected to increase due to climate change, which will negatively affect crop yields and food security (McClean et al., 2011). It should also be noted that the bean is very sensitive to both soil and environmental fluctuations which has made breeding for drought tolerance in the crop especially difficult. As a result, only a few breeding schemes have been successful in improving the efficiency of genetic enhancement of the common bean for drought tolerance.

Significant research efforts have been made, particularly over the past two to three decades, to improve common bean adaptation to drought and, as such, key drought genetic resources have also been identified. Genetic variation for most traits associated with drought tolerance has shown a quantitative inheritance (Asfaw et al., 2012; Blair et al., 2012). However, the underlying genetic basis of most of these traits is yet to be understood. Also, quantitative trait loci (QTL) underlying drought tolerance in the common bean have been discovered over the past two decades (Rao et al., 2006), although marker assisted selection using markers linked to these QTL is still uncommon. In the future, effective use of genomic tools will be efficient with a better understanding of the physiology of drought response and drought resistance mechanisms. Furthermore, Mesoamerican beans are the most widely grown beans around the world and are found in areas where drought stress is on the increase (Blair et al., 2012). As such, most QTL studies in drought tolerance have been carried out using Mesoamerican and intergene populations. Thus, more studies using Andean derived crosses are needed to explore additional diversity for drought resistance QTL alleles, and to analyze the effect of genetic backgrounds on the QTL alleles that have already been identified.

In common bean, molecular and protein markers were used to construct the first genetic linkage maps (Nodari et al., 1993). Amplified fragment length polymorphisms (AFLP), simple sequence repeats (SSR), resistance gene analogs (RGA) and single nucleotide polymorphic markers (SNP) have since been added to increase the density of existing maps. SNP markers are expected to provide the opportunity to produce high density maps enabling high precision QTL mapping in common bean. Limited drought studies in common bean have been conducted in the region and as a result marker-assisted breeding for drought tolerance has not yet been fully implemented. This could be due to the variability of drought stress, limited capacity for phenotyping, absence of high-throughput marker systems as well as the lack of knowledge of the genetics of drought resistance mechanisms. QTL analysis for drought tolerance across a range of environmental conditions using a dense map would improve the identification of QTL associated with broad adaptation to drought stress in common bean.

The objective of this study was to identify QTL underlying drought tolerance in the Andean cross of (BRB 191×SEQ 1027) RIL population. More studies in this field would greatly contribute to breeding programs through marker-assisted selection and broaden our understanding of the mechanisms behind drought tolerance, a key to averting production losses due to drought. In the near future, specific breeding strategies have to be developed to address all kinds of drought.

## MATERIALS AND METHODS

### The mapping population

An intra-gene pool RIL population consisting of 128 lines was derived from a cross between BRB 191 and SEQ1027, two Andean cultivars with bush growth habit. BRB 191 is the source of the *bc3* gene that confers resistance to Bean Common Mosaic Virus (BCMV) and its necrotic strain Bean Common Mosaic Necrosis Virus (BCMNV) while SEQ 1027 is a drought tolerant cultivar developed at CIAT (CIAT, 2008). The population was developed at CIAT, Cali by artificial hybridization to create an initial F_1_ hybrid followed by single seed descent (SSD) from the F_2_ up to the F_5_ generation. CIAT-Cali lies at an altitude of about 965 m with latitude 3°30'N and longitude of 76°30'W. The average temperatures are 24.3°C ranging from 18.8 to 28.4°C. Average rain fall is 896 mm and soils are fine silty, mixed, isohyperthermic Aquic Hapludoll. The population was evaluated along with eight experimental checks using incomplete block alpha-lattice design replicated twice. The evaluation was carried out under field conditions for 2 years and greenhouse conditions for 1 year at the National Agricultural Research Laboratories (NARL), Kawanda. The checks included Diacol calima, DAB 494, SEQ 1003, SCR 9, DAB 441, NABE 4, CAL 96 and BAT 477. Each experiment consisted of two water regimes treatment, non-stress (NS) where the plants were irrigated when there was no rain, and drought stress (DS) where there was no irrigation as described by Nabateregga et al. (2018).

In the greenhouse, soil was collected from a bean growing field site and mixed with sand to form a soil: sand mixture in a 2:1 proportion by weight (Polania et al., 2012). Sand was included to induce drought treatment faster than with soil alone. The soil was fertilized with adequate level of nutrients using NPK. The soil-sand mixture was poured into plastic and transparent 2 L pots of 15 cm diameter and 10 cm height with 3 small holes at the bottom to allow drainage. The pots were filled to the brim with the soil-sand mixture. Seeds were sterilized with a solution of 5% calcium hypochlorite for 5 min to reduce exposure to bean disease pests and placed on germination paper under ambient conditions for 2 days before planting (Makunde, 2013).

### Phenotypic data collection and analysis

During the phenotypic evaluation, 18 phenological, morphological and physiological traits were measured (Nabateregga et al., 2018). Drought intensity index (DII) was computed in each given environment as: 1-(Xs/Xi) (Fischer and Maurer, 1978); where Xs is the grand mean yield of all genotypes grown under drought stress and Xi is the grand mean yield of all genotypes grown under optimum conditions.

Histograms were drawn to observe the distribution of the genotypes means averaged across the two years under each water regime treatment. Pearson correlation analysis was also conducted to determine the linear relationship between the phenotypic traits evaluated under the two water treatments conditions herein presented as heat-map. The phenotypic data analysis was carried out using the R open source statistical software version 3.5.1.

### Genetic mapping and QTL analysis

A bulk leaf tissue sample of three F5 plants collected from seedlings of each cultivar in the population was used for DNA extraction. The population, along with the parents, was genotyped using the BARCBean6K-3 SNP array which contained a total of 5398 bead-types (Michigan State University, 2012) at KBiosciences. A linkage map was constructed using QTL ICIMapping software (Version 4.0, www.isbreeding.net; Jiankang et al., 2014). This was done after data from SNP genotyping of the population was manually inspected in an excel sheet to eliminate SNPs with no call or those which were monomorphic between parents. Besides, SNPs markers with distorted segregation were also checked and excluded from the map construction using Chi-square goodness-of-fit test at 5% significance level. The map was generated using the maximum likelihood mapping algorithm of QTL ICIM 4.0 and linkage groups were determined at a Logarithm of Odds (LOD) score of 3.0 and a maximum distance of 50 cM using the Haldane units (Lander et al., 1987). QTL analysis was conducted using composite interval mapping (CIM) in the ICI-Mapping software (Zhang et al., 2012) with 1 cM walk speed, a 0.001 PIN and 3.0 LOD significant threshold. The marker within the QTL peak with the highest phenotypic variation explained (PVE) and level of probability (P ≤ 0.01) were used to define the genomic position of the QTL. The analysis was done using the genotype means first for individual environments and then combined (average over the two years) in combined years analysis. To be reported, a QTL had to be detected in more than one environment and identified QTL were named according to the ‘Guidelines for common bean QTL nomenclature’ (Miklas and Porch, 2010). This consisted of combining a three-letter code for the trait with the linkage group and the order of the QTL for the given trait on each linkage group. For example, SY1.1^BS^ represents the first QTL for seed yield on chromosome Pv01 in the BRB/SEQ RIL population. Single environment analysis model was preferred to the multi-environment mixed model approach in order to detect repeatability, consistency and therefore usefulness of the QTL.

## RESULTS

### Drought stress effects on select phenotypic traits and correlation among traits

**Figure 1:**
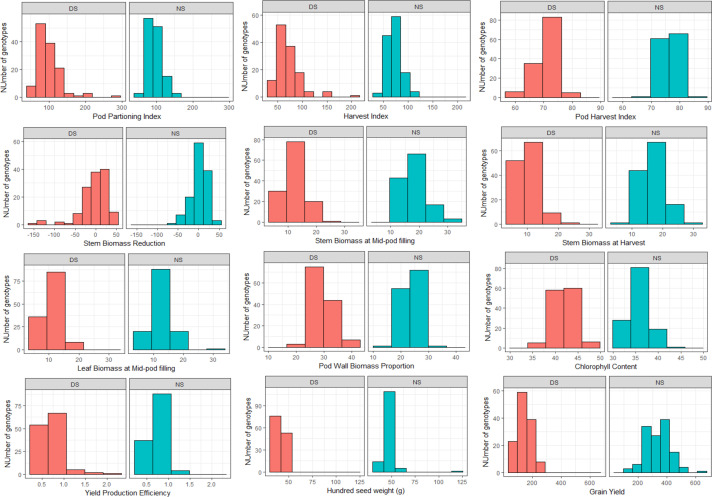
Distribution of BRB 191 × SEQ 1027 recombinant inbred lines’ means for select traits studied means under drought stress and non-stress conditions at Kawanda in 2014 and 2015.

Drought stress was the most severe during the field trial of 2014 with a drought intensity index (DII) of 0.78 followed by the greenhouse trial (DII of 0.58) while the 2015 field trial had the least index of 0.42. Overall, the drought stress affected the performance of the RILs for the various traits studied as shown by the distribution on the histogram (Figure 1). The RILs performed generally better under no stress (NS) conditions than drought stress (DS) for most of the traits; except for pod partitioning and harvest indices where the population had a relatively similar performance under both DS and ND (Figure 1). This was mainly noted for traits such as grain yield where under DS, none of the lines produced more than 300 kg/plot while under NS, about 40% of the RILs had a yield greater than 400 kg/plot. It was however noted that, under DS conditions, some lines performed equally good or better than the best performing ones under NS which is indicative of their resilience to water stress. This was the case for traits like pod partitioning index (PPI), yield production efficiency and chlorophyll content (Figure 1).

**Figure 2:**
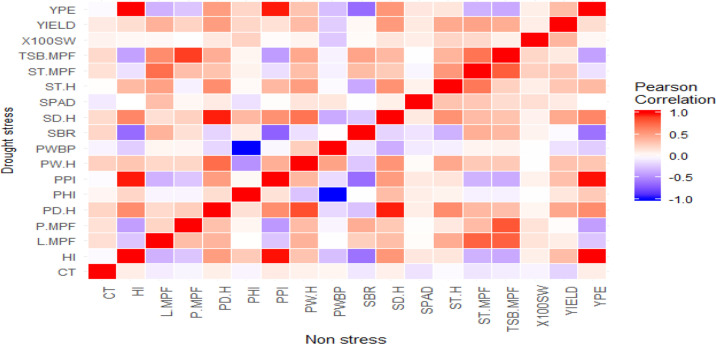
Heat map of Pearson correlation among traits studied under drought stress (below diagonal) and non-stress (above diagonal) conditions in 2014 and 2015 at Kawanda. YPE: Yield production efficiency; 100SW: hundred seed weight; TSB.MPF: total shoot biomass at mid-pod filling; ST.MPF: stem biomass at mid-pod filling; ST.H: stem biomass at harvest; SPAD: chlorophyll content; SD.H: seed biomass at harvest; SBR: stem biomass reduction; PWBP: pod wall biomass proportion; PW.H: pod wall biomass at harvest; PPI: pod partitioning index; PHI: pod harvest index; PD.H: pod biomass at harvest; P.MPF: pod biomass at mid-pod filling; L.MPF: leaf biomass at mid-pod filling; HI: harvest index; CT: canopy temperature.

A perfect positive correlation was observed among yield production efficiency (YPE), pod partitioning index (PPI) and harvest index (HI) on one hand and between seed and pod biomass on the other hand (Figure 2). On the contrary, a very strong negative correlation was observed between pod harvest index (PHI) and pod wall biomass proportion (PWBP).

With a specific focus on correlation between yield and other traits, 100 seed weight and yield production efficiency (YPE) had moderate and low positive correlations with yield under DS and NS, respectively (Figure 2). Harvest and pod partitioning indices (HI and PPI) had a positive correlation with yield which was moderate under NS but weak under DS unlike pod harvest index (PHI) which showed an opposite pattern with a weak correlation under NS but moderate under DS. In general, moderately high positive correlations were observed between the dry weight of various biomass components and yield under both DS and NS (Figure 2). Seed biomass at harvest (SD.H) had the highest correlation under DS. Canopy temperature showed moderate negative correlation with yield under NS and none under DS. Regardless of the water stress conditions, no correlation was observed between yield and stem biomass reduction (SBR) and chlorophyll content (SPAD) (Figure 2).

### Identification of quantitative trait loci for drought tolerance

The binning tool in ICIM software (version 4.0) removed redundant markers and those with high missing rates to produce 53 markers that were used to construct the linkage map of 11 anchor groups. Map saturation was low with three (pv5, pv6 and pv7) of the genetic groups carrying only one marker (Table 1). Linkage groups pv10 and pv9 had the highest number of markers (19 and 11 markers, respectively). The BRB 191/SEQ 1027 map spanned 486.29 cM with the 9th anchor group having the longest span of 107.418 cM (Table 1). The interval between markers for the 11 anchor groups ranged from 0.19 to 82.39 cM with an average distance of 12.03 cM between markers. The 11 linkage groups represented 40.52% limited genome coverage of common bean that has an estimated total size of 1200 cM.

**Table 1:** Anchor groups of the linkage map with the number of flanking markers and distance for each chromosome.

Chr ID	Anchor group	Number of SNP markers	Length (cM)
Pv01	1	3	41.221
Pv02	2	2	32.93
Pv03	3	3	30.198
Pv04	4	6	92.545
Pv05	5	1	0
Pv06	6	1	0
Pv07	7	1	0
Pv08	8	3	61.546
Pv09	9	11	107.418
Pv10	10	19	81.861
Pv11	11	3	38.571
Whole Genome		53	486.29

Chr ID: Chromosome identity.

**Figure 3:**
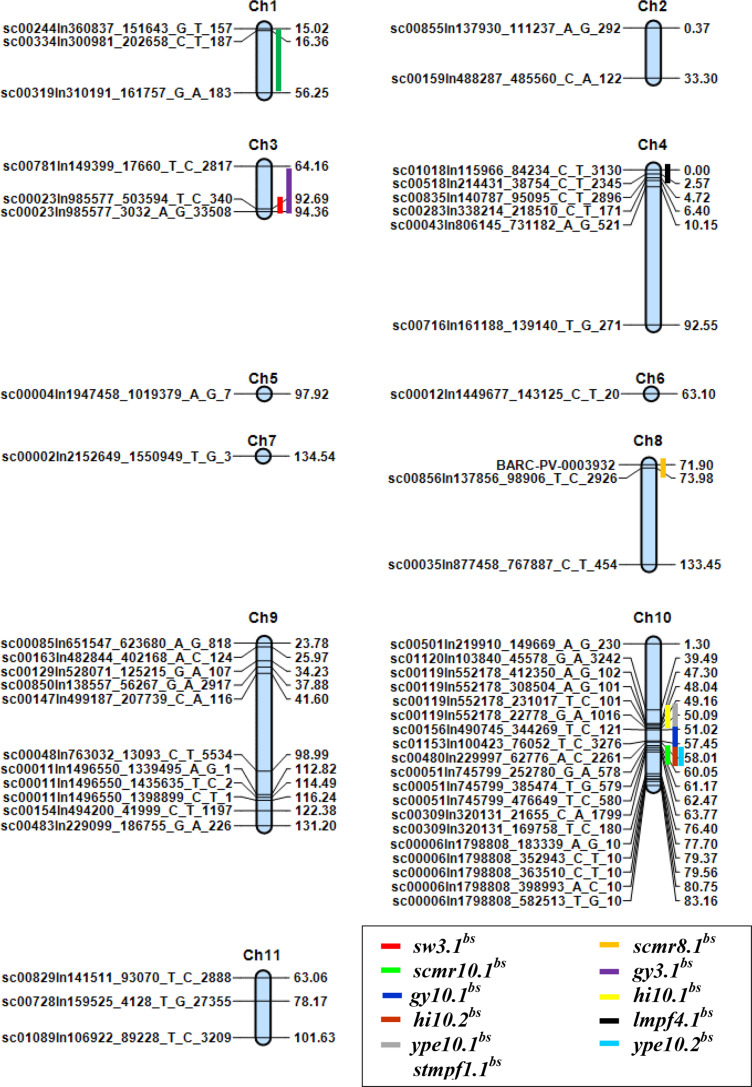
Linkage map of the BRB 191 × SEQ 1027 population and the QTLs identified Constructed using QTL ICI-Mapping software version 4.0 (Wang et al., 2014).

Inclusive composite interval mapping (ICIM) identified eleven major QTLs (LOD ≥ 3.00, P<0.05) from all experiments. All major QTL were located on 5 linkage groups, namely pv01, pv03, pv04 and pv08 and pv10 (Table 2); linkage group 10 (pv10) had the highest number of major QTLs (6) while pv08, pv04 and pv01 had only one. A major ‘hot spot’ was identified on pv10 where six QTLs clustered between position 40 and 60 cM (Figure 3). Consistent QTLs were detected for grain yield on pv03 and pv10 (*gy3.1^bs^* and *gy10.1^bs^*). Both QTLs were adaptive in nature being detected under non-stress conditions of 2015 and combined environments and they originated from BRB 191. The percentage of variation explained (PVE) for *gy10.1^bs^* was equal in both environments (9%) with comparable additive effects while *gy3.1^bs^* explained 4.9 to 7.2% percentage increase in seed yield on pv03. There was co-localization between the QTLs *sw3.1^bs^* related with seed weight and *gy3.1^bs^* related with grain yield on pv03 at 94.2 cM. However, the latter was detected under non-stress conditions of 2014 while the former under drought stress in 2014.

**Table 2:** Major QTL identified by ICIM from phenotypic data from field and greenhouse evaluation at Kawanda.

Trait of association	Environment	Treatment	Chr	QTL	QTL peak position (cM)	QTL interval (cM)	LOD	PVE(%)	Additive effect
	Field	Combined_DS	3		94.2	93.7-94.2	4.6	21.2	1.5
100 Seed weight	Field	Combined_NS	3		94.2	92.7-94.2	4.3	0.2	2.0
	Field	DS14	3	*sw3.1^bs^*	94.2	92.7-94.2	3.8	12.3	1.9
	Field	DS15	3		94.2	92.7-94.2	3.2	15.1	1.4
	Field	NS15	3		94.2	92.7-94.2	3.7	1.1	1.3
	Field	Combined_DS	8		71.9	71.9-72.4	6.5	21.3	-0.9
				*scmr8.1^bs^*					
	Field	DS14	8		71.9	71.9-72.4	6.1	22	-1.5
Chlorophyll content									
	Greenhouse	DS_GH	10		58.3	57.8-58.8	27.2	16.7	-2.0
				*scmr10.1^bs^*					
	Greenhouse	NS_GH	10		58.3	57.8-59.8	5	1.7	-0.9
	Field	Combined_NS	3		89.2	78.7-94.2	4.7	4.8	-32.8
	Field	NS15	3	*gy3.1^bs^*	94.2	92.7-94.2	3.2	7.2	-38.1
Grain yield									
	Field	Combined_NS	10		52.3	49.8-57.8	9.9	9	-45.2
	Field	NS15	10	*gy10.1^bs^*	57.3	50.8-57.8	4.1	9	-42.8
	Field	Combined_NS	10		41.3	19.8-45.8	4.2	1.3	5.6
				*hi10.1^bs^*					
	Field	NS15	10		42.3	39.8-45.8	4.3	7.4	9.1
Harvest index									
	Field	Combined_NS	10		62.3	60.8-63.8	3	0.8	-4.4
				*hi10.2^bs^*					
	Field	DS14	10		62.3	60.8-62.8	11.8	5.7	22.1
Leaf Biomass at MPF	Field	Combined_NS	4		0.0	0-2.5	3.2	3.7	-1.0
	Field	NS15	4	*lmpf4.1^bs^*	0.0	0-2.5	3.5	6.5	-1.4
Stem Biomass at MPF	Field	DS14	1		42.0	26.5-56	3.4	10.6	-1.6
	Field	Combined_DS	1	*stmpf1.1^bs^*	50.0	29.5-56	3.5	11.9	-1.7
	Field	Combined_NS	10		41.3	19.8-45.8	4.2	1.3	0.1
Yield Production Efficiency	Field	NS15	10	*ype10.1^bs^*	42.3	39.8-45.8	4.3	7.4	0.1
									
	Field	Combined_NS	10		62.3	60.8-63.8	3	0.8	0.0
				*ype10.2^bs^*					
	Field	DS14	10		62.3	60.8-62.8	11.8	5.7	0.2

Chr: Chromosome, Position: marker position in cM on the chromosome, LOD: logarithm of odds, PVE (%): phenotypic variation explained, DS: drought stress, NS: non-stress.

The QTL associated with 100 seed weight (*sw3.1^bs^*) was the most consistent; appearing in five environments namely drought stress (DS) conditions of 2014 and 2015, non-stress (NS) conditions of 2015 and combined environments of DS and NS. This constitutive QTL was detected on pv03 between 92.7 and 94.2 cM and expressed its highest additive effects under combined non-stress conditions (Table 2). Other consistent QTL detected in this study included those associated with chlorophyll content (*scmr8.1^bs^* and *scmr10.1^bs^*) on pv08 and pv10, harvest index (*hi10.1^bs^* and *hi10.2^bs^*) on pv10, dry weight of stem biomass at mid-pod filling (*stmpf1.1^bs^*) on pv01 and dry weight of leaf biomass at mid-pod filling (*lmpf4.1^bs^*) on pv04.

Both *scmr8.1^bs^* and *scmr10.1^bs^* originated from the female parent (BRB 191), however, the former was adaptive in nature (detected under drought stress conditions only) while the latter was constitutive being detected under both drought stress and non-stress in greenhouse. *scmr8.1^bs^* had a PVE ranging from 21.3 to 22% while *scmr10.1^bs^* increased chlorophyll content by a percentage that ranged from 1.7 to 16.7% (Table 2). The two QTLs for harvest index *hi10.1^bs^* and *hi10.2^bs^* were both consistent with the former being adaptive to non-stress conditions of 2015 and combined environments. On the other hand, *hi10.2^bs^* was constitutive in nature appearing under drought stress conditions of 2014 and combined non-stress environments (Table 2).

Coming to dry mass components, two QTLs were detected namely *stmpf1.1^bs^* associated with stem dry mass at MPF on linkage group pv01 and *lmpf4.1^bs^* associated with leaf dry mass at MPF on pv04. *stmpf1.1^bs^* was adaptive to drought stress conditions and was detected between 26.5 to 56 cM explaining 10.6 to 11.9% increase in stem dry mass at MPF on pv01. Similarly, *lmpf4.1^bs^* was adaptive in nature, being detected under non-stress conditions. It originated from BRB 191 and was detected between 0 and 2.5 cM on pv04; explaining 3.7 to 6.5% increases in leaf dry mass.

## DISCUSSION

The labor intensity involved in phenotyping for drought tolerance has made marker assisted breeding (MAB) a desirable tool in drought tolerance breeding programs. This study aimed at identifying QTLs that can provide markers that could potentially be used in MAB. The efficiency of QTL mapping is highly dependent on marker density (Mukeshimana et al., 2014; Perez-Vega et al., 2010). The genetic map constructed in this study had a size of 486.29 cM developed using 53 anchored polymorphic SNPs markers of the KASP genotyping assay. The successful construction of a genetic map using an Andean intragene cross population was critical given that Andean beans are widely grown in Uganda where drought stress is on the increase (Kalyebara and Buruchara, 2008; Miller, 2014).

Several studies have identified drought related QTLs (Asfaw et al., 2012; Mukeshimana et al., 2014); however, stable expression of these QTL across different stress environments is still rare (Trapp et al., 2015). This is because quantitative traits are highly dependent on environmental conditions and therefore variations in drought stress (in terms of timing, duration and intensity) as well as other abiotic and biotic factors can influence expression of quantitative genetic factors (Blum, 2011). Furthermore, the exclusive use of bi-parental mapping populations which enable only two alleles for any one gene to be evaluated (Xu and Crouch, 2008) also reduces the probability of detecting QTL consistently. In this study, QTLs were detected under both DS and NS conditions in 2014 as well as in 2015. About 70% of the QTLs identified were adaptive to either DS or NS while the other 30% were constitutive suggesting a high likelihood of consistent expression of the latter. Besides, half of the QTLs identified in this study originated from the male parent SEQ 1027 and this genotype was reported in previous studies as a superior Andean genotype for drought adaptation (CIAT, 2008; Asfaw, 2011) and as such the cultivar has been used in many breeding programs (Makunde, 2007).

The QTL analysis showed that the traits 100 seed weight, chlorophyll content, grain yield harvest index, leaf and stem dry mass and yield production efficiency are important to consider for selection for drought tolerance. Two genomic regions were found to be associated with grain yield on linkage groups pv03 and pv10 and these findings are consistent with previous research. Trapp et al. (2015), used Buster × Roza RIL population and detected a QTL on pv10 associated with yield (SY10.1) located at 39.9 cM in close vicinity of *gy10.1^bs^* reported in this study at 49.3 cM. In another study on pinto bean population, Hoyos-Villegas et al. (2015) reported three QTLs associated with seed yield of which one was on Pv03 in proximity with *gy3.1^bs^* of this study. Similar to the present results, Blair et al. (2012) also discovered yield-associated QTL on pv03 and pv10 adaptive to non-stress and drought stress conditions, respectively. However, unlike Yld10.1 in his study, *gy10.1^bs^* is adaptive to non-stress condition though they both had relatively equal additive effects of 56.1 and 51.4, respectively. Wright and Kelly (2011) also identified two QTL for seed yield on pv10 and on Pv03 using a RIL population of black bean parents Jaguar and 115M. Blair et al. (2006) also found two small-effects QTL on Pv03 from two-season data using a population derived from a cross between ICA Cerinza and G24404 (red bean). Miklas et al. (2007) reported a QTL for yield on Pv03 in two locations in different years in a RIL population from the cross between Aztec and ND-88-106-04. There are variations in findings between this study and previous studies; however, this could arise from differences in environment, marker technologies and populations used. Nevertheless, there is strong indication that pv03 and pv10 carry genetic factors conditioning seed yield.

A QTL for 100 seed weight was exclusively mapped in one region on pv03 (*sw3.1^bs^*) and originated from SEQ 1027 parent. It was the most consistent in the study and was detected under both DS and NS conditions, which implied that genes on this chromosome might have a constitutive mechanism that conditions seed weight. Consistency in expression of *sw3.1^bs^* suggests higher heritability and stability; highlighting the importance of seed weight in marker assisted breeding (Collins et al., 2008). It explained up to 21.2% of increase in seed weight under DS conditions and also co-localized with seed yield QTL *gy3.1^bs^*. Negative linkages of seed weight and seed yield potential in common bean has been and is still problematic in bean breeding (Beaver and Osorno, 2009); however, positive correlation observed in this study provide opportunity for simultaneous selection for these two traits in Andean intra-gene crosses. Previous studies have also identified QTL for seed weight on pv03. In a study aimed at identifying QTL for root architecture traits correlated with phosphorus acquisition, Beebe et al. (2006) detected Swf3.1 associated with SW using a cross between Andean G19833 and Mesoamerican DOR 364. Mukeshimana et al. (2014) also identified an important constitutive QTL for seed weight on pv03 (SW3.1SC) that explained 8 to 16% of the phenotypic variation. Similar to *sw3.1^bs^*, SW3.1SC was associated with yield under NS conditions. The seed weight QTL *sw3.1^bs^* also confirms results from various previous studies that mapped seed weight on Pv03 using RFLP and RAPD markers (Nodari et al., 1993; Park et al., 2000). Most recently, Sandhu et al. (2018) used 80,398 SNP markers on a cross between black bean lines BK004-001 and H68-4 and mapped QTL for seed weight on three linkage groups including pv03.

Repeatability of QTL across environments is an indicator of stability and increased heritability which improves efficiency of traits in marker-assisted breeding. Selecting for drought tolerance using constitutive QTL such as *sw3.1^bs^* in common bean under drought stress would favorably increase yield under non-stress conditions as well. Another important trait with a constitutive QTL was harvest index on pv10 (*hi10.2^bs^*) that explained 6% of the phenotypic variation. The second QTL for harvest index, *hi10.1^bs^* was adaptive to non-stress conditions. Using composite interval mapping, Asfaw et al. (2012) also reported QTLs for harvest index of which one was mapped on pv10 under non-stress conditions with a percentage variation of 0.12 to 0.23% similar to *hi10.1^bs^*. The stability of expression of *hi10.2^bs^* QTL under both drought stress and non-stress conditions is an indicator that marker assisted breeding using markers tightly linked to HI could possibly increase drought tolerance across a wide range of environments. Several studies have shown improved remobilization of photosynthate to grain to be an important mechanism under drought stress (Asfaw et al., 2012; Mukeshimana et al., 2014; Trapp et al., 2015); however, pyramiding different tolerance mechanisms will help develop a more sustainable drought adaptation in common bean.

In this study, we have also identified two QTLs associated with the dry weight of leaf and stem biomass (*stmpf1.1^bs^* and *lmpf4.1^bs^*) on pv01 and pv04, respectively. Similar findings were reported by Briñez et al. (2017) who detected SBF1.1AS, a QTL associated with stem biomass, on linkage group 1. This QTL had the same percentage variation explained as *stmpf1.1^bs^* (PVE =10.26%) highlighting the important role of this genomic region in the expression of drought tolerance mechanism. Repinski et al (2012) also mapped the PvTFL1y gene that controls stem dry mass and ease of mechanized harvest on pv01 (linkage group 1) in common bean, and this further supports the present findings.

While in this study consistent QTLs associated with chlorophyll content were detected on pv08 and pv10 (*scmr8.1^bs^* and *scmr10.1^bs^*), on pv01, pv06 and pv11; these were inconsistently compared to the ones identified in this study that appeared in more than one environment. However, it should be noted that the percentage variations for chlorophyll content detected in the Briñez et al. (2017) study had higher percentage variations explained, ranging from 11.2 to 32.9% compared to the 1.7 to 21% reported in this present study. Briñez et al. (2017) used SSRs and SNP markers to map QTLs in a SEA 5 × AND 277 common bean cross. Asfaw et al. (2012) identified 13 QTL associated with chlorophyll content of which three were detected on pv08 and pv10 as found in this study. Similar to this study, these QTL detected on pv10 were constitutive like *scmr10.1^bs^* while those found on pv08 were adaptive in nature to drought stress conditions like *scmr8.1^bs^*. However, it should be noted that high chlorophyll content does not necessarily translate to higher yields. Some have reported that thick, small, and dark green leaves under drought might be less photosynthetically active because of closed stomata despite high chlorophyll content (Asfaw et al., 2012), and as such SPAD readings and QTL for this trait may be less useful under severe drought stress compared with moderate stress.

Finally, one of the highlights in this study was co-localization of QTL on pv10. Co-localization of drought-related factors on pv10 was an indication of pleiotropic effects on the chromosome (Aastveit and Aastveit, 1993) and confirmed the complexity of seed yield under drought stress, a phenomenon determined by many physiological processes throughout crop growth. Clustering of several related factors could also have been because these traits are physiologically related and may have a common biochemical pathway. Co-localization of QTL for correlated variables such as phenology, yield, and yield components on the same chromosome has also been reported in other studies (Mukeshimana et al., 2014; Trapp et al., 2015; Briñez et al., 2017).

In conclusion, the present study identified 11 important constitutive and adaptive drought tolerance-associated QTLs in the BRB 191 × SEQ 1027 RIL population. These results agree with previous studies which mapped drought related QTLs in similar chromosome regions thus demonstrating the merits of QTL mapping using SNP technologies as a precision tool in genetic studies. New regions containing novel QTLs were also identified in this study for drought related traits such as stomatal conductance, biomass accumulation and photosynthate remobilization. These results will help for fine mapping candidate genes controlling these traits hence an important tool that can be used for selection. The knowledge about these QTLs will also help foster marker-assisted selection in common bean breeding programs using SEQ 1027 as source of drought tolerance. In view of the complexity of seed yield under drought stress, and the inconsistency in the expression of trait associated QTL across environments and mapping populations, selection using a combination of drought related traits would produce more stable and high yielding genotypes.
